# Maladaptive exercise in eating disorders: lifetime and current impact on mental health and treatment seeking

**DOI:** 10.1186/s40337-024-01048-2

**Published:** 2024-06-24

**Authors:** Zhenxin Liao, Andreas Birgegård, Elin Monell, Stina Borg, Cynthia M Bulik, Emma Forsén Mantilla

**Affiliations:** 1 Department of Medical Epidemiology and Biostatistics, Karolinska Institute, Nobels väg 12a, Stockholm, 171 77 Sweden; 2 Stockholm County Council, Stockholms Centrum för ätstörningar, Wollmar Yxkullsgatan 27, Stockholm, 118 50 Sweden; 3 Department of Psychiatry, University of North Carolina at Chapel Hill, CB #7160, 101 Manning Drive, Chapel Hill, NC 27599-716 USA; 4 Department of Nutrition, University of North Carolina at Chapel Hill, Chapel Hill, NC 27599-716 USA; 5 The Swedish School of Sport and Health Sciences, GIH, Lidingövägen 1, Box 5626, Stockholm, 114 86 Sweden

## Abstract

**Background:**

Many patients with eating disorders report exercise as a central symptom of their illness—as a way to compensate for food intake, prevent weight-gain, and/or reduce negative affect. Previous findings show associations between maladaptive exercise and more severe eating disorder pathology, higher risk for relapse, other co-morbid symptoms, and worse treatment outcome.

**Methods:**

In this study, we included 8252 participants with eating disorders and investigated associations between maladaptive exercise (both lifetime and current) and ED pathology, illness duration, depression, anxiety, self-harm and suicidal ideation, and treatment seeking patterns in individuals with lifetime maladaptive exercise. Participants were included via the Swedish site of the large global study The Eating Disorders Genetics Initiative (EDGI) and completed measures of both lifetime and current symptomatology.

**Results:**

Results indicate that lifetime maladaptive exercise is associated with higher prevalence of lifetime depression and anxiety and with patients more often receiving treatment, although these results need to be investigated in future studies. Current maladaptive exercise was associated with more severe ED symptoms, and higher levels of depression, anxiety, obsessive-compulsive traits, and suicidal ideation.

**Conclusions:**

Our findings point to the complexities of exercise as an eating disorder symptom and the need for clearly assessing and acknowledging this, as well as tailoring interventions to treat this symptom to achieve sustainable recovery.

**Supplementary Information:**

The online version contains supplementary material available at 10.1186/s40337-024-01048-2.

## Abstract (plain language summary)

Many individuals with eating disorders view exercise as a crucial aspect of their illness, often using it to control weight and emotions. Research suggests that maladaptive exercise correlates with more severe eating disorder symptoms, increased risk of relapse, and poorer treatment outcomes. Analyzing data from 8252 participants with eating disorders, this study from the Swedish site of The Eating Disorders Genetics Initiative (EDGI) found that lifetime maladaptive exercise is linked to higher rates of depression and anxiety and increased treatment seeking behaviors. Current maladaptive exercise was associated with heightened eating disorder severity, depression, anxiety, obsessive-compulsive traits, and suicidal thoughts. These findings underscore the importance of recognizing exercise as a symptom of eating disorders and tailoring interventions accordingly for sustainable recovery.

**Supplementary Information:**

The online version contains supplementary material available at 10.1186/s40337-024-01048-2.

## Background

Eating disorders (EDs) are highly complex, often long-term conditions that impose significant emotional, psychological, and economic burden on individuals and society [1]. An already concerning prevalence [2, 3] was exacerbated during the COVID-19 pandemic, with a notable increase in the overall incidence of EDs [4–6]. The economic costs associated with EDs are substantial, with the average annual cost per patient ranging from $5,000 for binge-eating disorder (BED) to $18,000 for anorexia nervosa (AN) [1, 7]. Recognizing and treating central eating disorder (ED) symptoms at an early stage has the potential to improve outcomes, prevent long-term illness, and mitigate costs [8–10].

A large proportion of ED patients report being physically active and many identify exercise as a symptom of their illness; e.g. a way to compensate for food intake, prevent weight-gain, and/or reduce negative affect [11–13]. Various terms have been proposed for excessive and potentially harmful exercise. For present purposes, maladaptive exercise (ME) broadly denotes any exercise that leads to negative outcomes or disrupts daily functioning. Studies suggest that ME is associated with more severe ED pathology [14–16], longer in-patient treatment [17], and increased risk for relapse in AN [18]. The term compulsive exercise (CE) has gained popularity during the last decade in ED research [19–22]. It denotes exercise to reduce negative affect or prevent a feared outcome, that has become rigid and compulsive, is engaged in despite illness or injury and to the detriment of other previously valued activities, and where inability to exercise causes distress [20, 21]. The association between CE and depression and anxiety has been explored in a few studies with various samples and outcomes [23–25]. In a non-clinical population, CE was negatively associated with depressive symptoms [25], whereas in an adolescent ED population it was positively associated with anxiety [23]. Given the association between CE and emotion regulation, and ME with a more severe symptom pattern overall, studying associations with co-morbid symptoms (e.g. anxiety, depression, suicidal ideations) is of importance.

Despite its significance, exercise as a symptom has often been overlooked in ED research and clinical practice [26–28]. This oversight might be partly due to exercise being only vaguely described in current ED diagnostic criteria. Exercise is mentioned as a possible compensatory behaviour and as a means of preventing weight-gain, but there is no guidance in relation to inherent qualities that differentiate unhealthy from healthy physical activity [29]. Also, unlike behaviours such as vomiting, exercise is socially acceptable and encouraged in the general society to promote health. This may also contribute to difficulties in recognising exercise as a problematic behaviour for both healthcare professionals and the patients themselves. Further, to date no systematic evidence-based treatment specifically targeting ME/CE in EDs exists, meaning that even if the patient seeks treatment there is little to offer besides standardised EDs care [28]. There is a risk that individuals with exercise as a central symptom of their ED could go unrecognized for longer, be less likely to be referred to or receive treatment, and/or receive treatment that does not fully address their ED behaviours. Investigating potential long- and short-term associations between ME/CE and other symptoms in EDs may inform our understanding of this symptom and its correlates, which in turn may have implications for identification, prevention, and treatment efforts. There is further a need to learn more about treatment patterns for individuals where exercise is central, as they may go undetected or receive insufficient treatment for their problematic behaviour, which may indeed have detrimental consequences.

In the present study, we introduce two subcategories of ME. “Problematic Exercise (PE)” is a subtype of ME that in this study denotes a lifelong pattern. If a person has ever engaged in this form of exercise, they are considered to have PE. The term “Compulsive Exercise (CE)” is reserved to identify *current* behavior associated with ME. This term applies when an individual is presently exhibiting a pattern of exercise that fits the criteria for CE as described by others [20]. Lifetime PE, measured using items not fully encompassing the definition of CE, was defined as a compulsive need to exercise where inability to exercise causes anxiety or distress, a drive to control body shape or weight, continuing to exercise despite injury or illness, and adjusting eating habits when exercise is not possible. In practice, these aspects mimic CE closely, but we use the term PE to acknowledge the different measurement methods used (see below). Current ME conformed to the CE definition, and was measured using the Compulsive Exercise Test (CET) [30].

### Aims

In this study, we investigated both lifetime and current ME in a large ED sample. Our first aim was to study associations between lifetime PE and lifetime depression, anxiety, ED illness duration, suicidality, and self-harm. A second aim was to investigate whether current CE was associated with current ED symptoms, depression, anxiety, compulsivity, self-harm, and suicidality. A third aim was to investigate treatment patterns for individuals with PE; if this is a poorly recognized or neglected symptom due to insufficient treatment options, individuals presenting with PE might consume care in different ways (e.g., to a lesser degree) compared to those without PE. As this area is previously unstudied however, we did not formulate hypotheses.

## Method

### Participants

The current study was conducted using a subsample from the parent study: the Eating Disorders Genetics Initiative-Sweden (EDGI; https://edgi.se). EDGI is a multi-country study recruiting individuals with lifetime or current EDs as well as controls, to study both environmental and genetic risk factors involved in EDs [31]. In this study, cases at the Swedish site (EDGI-SE) recruited up until the 26th of May 2023 were included. Eligible participants were aged 16 and older living in Sweden, with access to a Swedish Bank ID (electronic personal identifier) and ability to understand Swedish. They all had a current or lifetime history of AN, BN, BED, atypical AN, atypical BN, or atypical BED as according to the Diagnostic and Statistical Manual, fifth edition [29]. The initial sample included a total of *N* = 9192 participants. after excluding 936 due to response period limitation (some questionnaires asked about the last 14 days, so individuals who paused the survey and logged back in after more than than were excluded), and 4 due to being under the age of 16, 8252 participants remained. For analyses of lifetime exercise (PE), 6378 participants met our inclusion criteria, and for current CE, 7260 participants met our criteria (see “Statistical analysis” for a description of how groups were formed).

### Procedure

The recruitment approach was multi-pronged with participants being recruited via previous studies, public outreach, the Swedish national quality register for EDs (Riksät), and the market research company IPSOS [32]. Participants recruited from previous studies and Riksät were sent an invitation to participate via e-mail or post. Public outreach included posts in social media channels; promotion on podcasts; magazine- and newspaper articles; information about EDGI in a large production on Swedish national television, advertisement in the member magazine of the largest EDs patient advocacy organization, and posters and brochures at several EDs clinics around the country. Individuals deciding to take part in EDGI, signed up via the official project website and after receiving the appropriate information they consented using BankID. After consent, participants provided updated contact details and then moved on to complete the online screening survey to determine history of EDs. Individuals screening as cases were never subject to exclusion, thus they all moved on to complete the main online surveys. The main survey captured current ED symptoms, ED-related quality of life, compulsive exercise, treatment history, and a range of health and mental health symptoms. Completing the full survey (including the screening part) takes about 50–60 min. After completing the main survey, participants were sent kits for saliva DNA sampling. However, the genomic data are not included in the present study, and thus this part of the procedure will not be described further. After fulfilling all steps above, participants were sent two cinema gift cards as a token of appreciation.

### Measures

#### Instruments

EDGI-SE includes 14 on-line surveys. The main battery is identical to that used at other international EDGI sites and has been described elsewhere [31], though EDGI-SE also included measures for BED, ARFID, night eating, and cultural adaptations of the Eating Disorder Treatment History questionnaire. In the present study the following instruments were used and will thus be described further: the Eating Disorders 100 000 Questionnaire (ED100K V.3) [31]; the Eating Disorder Examination Questionnaire (EDE-Q) [33]; the Compulsive Exercise Test (CET) [30]; the Eating Disorder Treatment History questionnaire [31]; the Patient Health Questionnaire-9 (PHQ-9) [34]; the Generalized Anxiety Disorder 7-item scale (GAD-7) [35]; the GLAD questionnaire [31]; and the Obsessive-Compulsive Inventory-revised (OCI-R) [36].

#### The ED100K V.3

Besides initial demographics, this self-report questionnaire assesses lifetime DSM-5 criteria for AN, BN, BED and other specified feeding and eating disorders (OSFEDs). It is based on the Structured Clinical Interview for Diagnostic and Statistical Manual of Mental Disorders, fifth edition (DSM-5) EDs (the SCID) [37] and has been successfully validated against the SCID [38]. For the purpose of the present study, diagnostic categorization, lifetime symptom presentation, height and weight (i.e. BMI), course and onset of illness, and demographic variables were extracted. Exercise-related items, presented in supplementary materials, were used to group participants into PE and non-PE groups (see below).

#### The EDE-Q V.6

The EDE-Q version 6.0 [33] is a 28-item 7-point likert scale self-report assessing current cognitive and behavioral ED pathology with higher scores indicating greater difficulties. The instrument is summarized in a Global scale score, which was grouped using cut-off score of 2.8 [39]. The behavioral items ask for frequency of common EDs behaviors (such as compulsive exercise and binge-eating) over the past 28 days. The EDE-Q has good psychometric properties [33, 40, 41].

#### The CET

This is a 24-item self-report questionnaire intended to assess core psychological, emotional, and behavioral aspects of CE specifically in the context of EDs. The CET is based on a cognitive-behavioral theoretical framework [1] in order to also inform about potential treatment [30]. Items are scored on a scale of 0–5 (never true-always). It generates a total scale score and scores for the five subscales: Avoidance and rule-driven behavior, Weight control exercise, Mood improvement, Lack of exercise enjoyment and Exercise rigidity. A clinical cut-off score of 15 has been recommended as indicating CE [16]. In EDGI, individuals who report not to exercise at all, are exempted from completing the CET. The CET has satisfactory psychometric properties [14, 16, 30].

#### The EDs treatment history questionnaire

This is a country-specific battery of items inquiring about treatment in relation to EDs, insurance reimbursement and economic burden from being ill. The treatment items have been adapted to reflect the healthcare system of the specific country [31]. In the present study, data from items asking about treatment consumption, age when in treatment and the types of treatments received (e.g. outpatient treatment, inpatient care at an EDs unit, visit to emergency room) were retrieved.

#### The PHQ-9

The PHQ-9 is a nine-item questionnaire screening for depression severity [34]. The items are based on the DSM-IV criteria for major depression, focuses on the last 2 weeks and are scored between “0” (not at all) and “3” (nearly every day). Items ask how often the respondent has been bothered by e.g.: “feeling down, depressed or hopeless” and “feeling tired or having little energy”. The instrument has displayed adequate psychometric properties in various populations [42, 43].

#### The GAD-7

This is a seven-item questionnaire screening for generalized anxiety disorder [35]. The items focus on the last 2 weeks and are scored on a 4-point scale where “0” is “not at all” and “3” is “nearly every day”. Items ask how often the respondent has been bothered by e.g.: “feeling nervous, anxious or on the edge” and “worrying too much about different things”. Item scores are summed to generate a severity score. The GAD-7 is a reliable and valid instrument for assessing anxiety [35, 44, 45].

#### The GLAD questionnaire

This self-report measure was developed as part of the large GLAD study (https://gladstudy.org.uk) and consists of additional items assessing lifetime major depressive disorder (17 items) and lifetime anxiety disorders (21 items). The items resemble DSM-5 diagnostic criteria for the disorders and are phrased to capture lifetime experiences: i.e. “have you ever…” [31].

#### The OCI-R

This is an 18-item self-report measure inquiring about obsessive and compulsive symptoms over the past month [36]. Items are scored on a scale ranging between 0–4 (0=”not at all” and 4= “extremely”) with higher scores indicating greater difficulties. The OCI-R results in a total scale score and six subscale scores: washing, obsessing, ordering, checking, neutralizing, and hoarding. The instrument has demonstrated satisfactory psychometric properties in several studies [36, 46, 47].

### Statistical analysis

#### PE and CE group definitions

The PE group was characterized by ED100K responses suggesting (1) “often” having felt a compulsion to exercise coupled with feelings of anxiety or worry when unable to engage in physical activity (score of 3 on the “icb_exercise” item); (2) feeling an irresistible urge to exercise for the purpose of controlling body shape or weight (score of 1 on “ex_compel”); and (3) at least two of the following: experiencing anxiety or distress when prevented from exercising, continuing to exercise despite injuries or illnesses that would typically discourage others, and altering diet or eating patterns in response to the inability to exercise (combined score ≥ 2 on “ex_distress”, “ex_ill”, and “ex_diet”). The Non-PE group was defined by a score of 1 on “icb_exercise”, indicating that individuals in this category “never” feel compelled to exercise nor do they experience anxiety or worry when they are unable to perform physical activity. Participants scoring 2 on “icb_exercise” (denoting “Sometimes, but it never became a habit”) were excluded to achieve a clear theoretical contrast between those with and without lifetime PE. For analyses of current exercise, we used CET score cutoff, where CE scored ≥ 15 and non-CE < 15.

#### The definition of compensation

Lifetime compensatory behavior was defined as responses of “A few times, but it didn’t become a habit” or “Often” on the ED100K items inquiring lifetime use of laxatives, diuretics, fasting, dietpills and fasting (“icb_laxatives”, “icb_diuretics”, “icb_fasted”, “icb_dietpills”, and “icb_vomit”). Current compensatory behavior was captured as “Yes” responses to “vom_current” for vomiting, “lax_current” for laxatives, “wp_current” for diuretics, “fast_current” for fasting, and “dp_current” for diet pills. “No” to all of these was scored as no current compensation, and participants responding “Not sure” to any of the items, without any “Yes” responses, were excluded from the analysis.

#### The definition of suicidal ideations

Lifetime suicidal ideations was defined using the GLAD-item “do you think about death a lot or feel that it would be better if you were dead?”, and current suicidal ideations using item nine of the PHQ-9. A response of “never” indicates no ideations, while responses indicating ideations occurred on “several days,” “more than half the days,” or “nearly every day” were classified as symptomatic.

Demographic and background clinical variables were analyzed using chi-square or *t*-tests. To explore the relationship between lifetime psychological symptoms and exercise, we conducted a multinomial logistic regression with PE vs. Non-PE a dependent variable and lifetime depression, anxiety, and suicidality as independent. Additionally, we used a chi-square test to examine differences between the two groups in having received ED treatment. Age, gender, and current BMI were examined as possible covariates using the Breslow-Day-Tarone test to assess potential heterogeneity across strata. In CE vs. Non-CE analyses of current exercise impact, we used chi-square to compare current EDs illness status (i.e. EDE-Q global score < or > = 2.8), and ANCOVA for depression (PHQ9), anxiety (GAD-7), and obsessive-compulsive traits (OCI-R), and suicidal ideation (based on item 9 of the PHQ9), using age, current BMI, and gender as covariates. Sensitivity analyses were conducted excluding participants with AN and re-calculating all analyses, since most research on exercise has been done on this group, and we sought to investigate whether participants with a history of AN disproportionately contributed to any observed effects. Effect sizes were calculated as Cohen’s d (small ≥ 0.20; moderate ≥ 0.50; large ≥ 0.80) and Phi (small ≥ 0.10; moderate ≥ 0.30; large ≥ 0.50). Data were managed in Python version 3.9.6. Analyses and figures were generated using Python version 3.9.6 and IBM SPSS 27.

## Results

### Demographic and clinical characteristics

Table 1 presents background variables for the PE groups. PE participants were more likely to be female, and they more often had AN, whereas Non-PE participants were more likely to have BN, BED, or OSFED. Further, more PE participants reported compensatory behaviors, were younger both when they experienced their first EDs symptom and when they participated in the study, had lower BMIs, and were more likely to have undergone ED treatment. Sensitivity analysis excluding AN participants did not substantively change the results (Table S1).

**Table 1:** Demographic and clinical characteristics for the Non-PE and PE groups (including AN)

Category		*N* (column %) or M ± SD		Total	χ^2^ or *t*	*p*
		Non-PE	PE			
Gender	Male	44 (6.21)	141 (2.49)	185 (2.90)	33.60	< 0.001
	Female	652 (91.96)	5456 (96.24)	6108 (95.77)		
	Non-binary	11 (1.55)	66 (1.16)	77 (1.21)		
	Others	2 (0.28)	6 (0.11)	8 (0.13)		
Diagnosis	AN	246 (39.05)	4207 (76.49)	4453 (72.64)	679.92	< 0.001
	BN	90 (14.29)	648 (11.78)	738 (12.04)		
	BED	81 (12.86)	62 (1.13)	143 (2.33)		
	OSFED	213 (33.81)	583 (10.60)	796 (12.99)		
Compensation		592 (83.50)	5381 (94.92)	5973 (93.65)	136.34	< 0.001
Current ED		268 (37.80)	2267 (39.99)	2535 (39.75)	1.26	0.261
EDs treatment		409 (57.69)	4778 (84.28)	5187 (81.33)	293.52	< 0.001
Age		39.60 ± 13.48	31.18 ± 10.11	32.11 ± 10.87	16.08	< 0.001
Age at first symptom		18.02 ± 6.68	15.28 ± 4.26	15.59 ± 4.67	10.63	< 0.001
Current BMI		27.74 ± 8.35	22.76 ± 4.70	23.31 ± 5.46	15.58	< 0.001
EDE-Q score		2.25 ± 1.49	2.35 ± 1.62	2.34 ± 1.61	-1.60	0.11

Note. PE = problematic exercise; AN = anorexia nervosa; BN = bulimia nervosa; BED = binge eating disorder; OSFED = other specified feeding or eating disorder; EDs = eating disorders; BMI = body mass index; EDE-Q = eating disorders examination questionnaire. Total *N* for different variables vary between 6130 and 6378 due to data exclusions described above

Table 2 shows background variables for the CE and Non-CE groups. There was no gender difference and no difference in current age or age at first ED symptom. CE participants were however more likely to report compensatory behaviors, they had lower BMI, higher EDE-Q Total score, and much more often reported a current ED. Again, excluding AN participants resulted in similar findings, except in relation to “age” which was significantly lower in the CE group without AN participants (Table S2).

**Table 2:** Demographic and clinical characteristics of Non-CE group and CE groups (including AN)

Category		*N* (column %) or M ± SD		Total	χ^2^ or *t*	*p*
		Non-CE	CE			
Gender	Male	161 (2.9)	50 (3.1)	211 (2.9)	0.68	0.878
	Female	5410 (95.8)	1547 (95.8)	6957 (95.8)		
	Non-binary	67 (1.2)	16 (1.0)	83 (1.1)		
	Others	7 (0.1)	2 (0.1)	9 (0.1)		
Diagnosis	AN	3770 (69.9)	1131 (72.0)	4901 (70.4)	22.58	< 0.001
	BN	681 (12.6)	213 (13.6)	894 (12.8)		
	BED	174 (3.2)	17 (1.1)	191 (2.7)		
	OSFED	766 (14.2)	210 (13.4)	976 (14.0)		
Compensation		1405 (29.0)	785 (55.7)	2190 (35.0)	372.24	< 0.001
Current EDs		1682 (29.8)	1226 (75.9)	2908 (40.1)	1112.28	< 0.001
EDs treatment		4536 (80.3)	1246 (77.2)	5782 (79.6)	7.94	0.005
Age		32.23 ± 10.93	32.32 ± 10.95	32.25 ± 10.94	-0.30	0.762
Age at first symptom		15.63 ± 4.48	15.52 ± 5.20	15.60 ± 4.65	0.78	0.438
Current BMI		23.52 ± 5.47	22.87 ± 5.50	23.37 ± 5.48	4.22	< 0.001
EDE-Q Total		1.97 ± 1.46	3.71 ± 1.31	2.35 ± 1.60	-46.00	< 0.001

Note. CE = compulsive exercise; AN = anorexia nervosa; BN = bulimia nervosa; BED = binge eating disorder; OSFED = other specified feeding or eating disorder; EDs = eating disorders; BMI = body mass index; EDE-Q = eating disorders examination questionnaire. Total *N* for different variables vary between 6259 and 7260 due to data exclusions described above

### Comparison of lifetime psychological factors between non-PE and PE groups

A multinomial logistic regression analysis was performed, with age, gender, and current BMI as covariates, to examine the association between lifetime psychological factors and the two distinct exercise groups. There were significant differences in all factors, with the PE group more often reporting lifetime depression (74.3% in PE vs. 70.4% in Non-PE; OR = 1.31 (95% CI = 1.09 ∼ 1.57, *p* = .005; Phi = 0.028), lifetime anxiety (46.9% in PE vs. 38.9% in Non-PE; OR = 1.33 (95% CI = 1.12 ∼ 1.58, *p* = .001; Phi = 0.050), and suicidal ideation (65.4% in PE vs. 57.1% in Non-PE; OR = 1.32 (95% CI = 1.10 ∼ 1.59, *p* = .003; Phi = 0.068). Although non-significant, sensitivity analyses showed a different pattern; lifetime depression (71.49% in PE vs. 72.57% in Non-PE; OR = 1.19 (95% CI = 0.92 ∼ 1.53, *p* = .191; Phi = 0.010), lifetime anxiety (38.01% in PE vs. 42.61% in Non-PE; OR = 1.24 (95% CI = 0.98 ∼ 1.56, *p* = .078; Phi = 0.040), and suicidal ideation (45.14% in PE vs. 51.30% in Non-PE; OR = 1.29 (95% CI = 1.03 ∼ 1.63, *p* = .028; Phi = 0.063).

### EDs treatment history between Non-PE and PE groups

A significantly higher proportion of the PE group (84%) had undergone treatment for EDs compared to Non-PE (58%); χ² = 293.524, OR = 3.933 (95% CI = 3.334–4.640, *p* < .001). Regarding types of treatment (Table 3), a higher proportion of the PE group had received all types of treatment, except outpatient- and emergency room visits; however, effect sizes were less than small (all ≤ 0.076). When removing AN cases (Table S3), the only significant difference remaining was for intensive outpatient treatment, although the overall pattern with a higher proportion of the PE group having received the different treatment types, remained. Again, effect sizes were less than small (all ≤ 0.076). Comparisons of therapy orientations (Table S4) showed that the PE group had significantly more often received cognitive-behavioral, psychodynamic, and physiotherapy as well as overall psychotherapy and group therapy. When removing the AN cases, however, the only difference remaining was that the PE group had received cognitive behavioral therapy more often (Table S5). Significant differences were not found for interpersonal or dialectical behavior therapy, nor for family counseling. Further, we found no difference between PE and Non-PE regarding how difficult they found it to access adequate treatment for their ED (Table S6).

**Table 3:** Comparison of treatment and types for EDs between the Non-PE group and PE groups

Type of treatment	*N* (column %)		Total	χ^2^	*p*	Phi
	Non-PE	PE				
Medical ward inpatient care	14 (3.6)	393 (8.4)	407 (8.1)	11.51	0.001	0.048
Psychiatric inpatient care	33 (8.4)	715 (15.4)	748 (14.8)	13.70	< 0.001	0.052
Inpatient care at an eating disorder unit	37 (9.5)	760 (16.3)	797 (15.8)	12.79	< 0.001	0.050
Care in treatment homes	17 (4.4)	424 (9.1)	441 (8.7)	10.26	0.001	0.045
Day treatment/partial hospitalization	74 (18.9)	1496 (32.1)	1570 (31.1)	29.40	< 0.001	0.076
Intensive outpatient treatment	57 (14.6)	1190 (25.6)	1247 (24.7)	23.42	< 0.001	0.068
Outpatient treatment	278 (71.1)	3311 (71.1)	3589 (71.1)	< 0.01	0.986	< 0.001
Emergency room visit	23 (5.9)	690 (14.8)	713 (14.1)	2.79	0.095	0.069
Others	88 (22.5)	886 (19.0)	974 (19.3)	23.77	< 0.001	0.023

Note. PE = problematic exercise

### The association between current CE and current EDs

Lifetime PE and current CE were significantly associated (Table 4). Among CE participants, 98% also reported lifetime PE, but a large majority Non-CE also reported lifetime PE. Further, we compared those who were currently engaged in CE with those who were not regarding current EDs status (Table 5). A notably lower proportion of Non-CE rated themselves as having current EDs (42.6%) compared to those with CE (80.6%). Also, a lower proportion of Non-CE scored above cutoff on the EDE-Q (29.8%) compared to the CE group (75.9%). Interestingly also, many who thought they currently had EDs (50.8%) did not score above the clinical cut-off point on the EDE-Q (40.1%). This discrepancy appeared larger in the Non-CE group; there was little difference between Yes and No in self-rated current ED (both around 42%); but on the EDE-Q, only 30% scored above the clinical cut-off. Corresponding proportions for the CE group were 81% Yes vs. 8% No in self-rated data, and inversely from the Non-CE group, 76% scored above the clinical cut-off on the EDE-Q and 24% below. Thus, the CE participants’ self-assessments were better calibrated with their EDE-Q scores, compared to the Non-CE group. The “Not sure” option for self-rated status was used in roughly similar proportions in both groups. Further, we compared responses to the EDE-Q item concerning exercise, asking whether they had “exercised excessively or compulsively to control your weight, figure, amount of fat, or to burn calories?” in the past 28 days, and found that it was significantly associated with CE group (χ2 = 1274.768, *p* < .001; Table S7). In the CE group, 30.7% responded negatively to this item (compared to 77.9% of Non-CE), whereas 69.3% of them did report such behavior (compared to 22.1% of Non-CE). Thus, CET-based grouping was significantly associated with responses to this item, although not perfectly so. Excluding AN participants (Tables S2, S8 and S9) yielded similar, albeit slightly less marked, patterns.

**Table 4:** Crosstabulation and chi-square of current CE and lifetime PE

		CE (column %)		Total	χ2	*p*
		Non-CE	CE			
PE	Non-PE	448 (10.4)	29 (2.0)	477 (8.3)	100.91	< 0.001
	PE	3844 (89.6)	1417 (98.0)	5261 (91.7)		
Total		4292	1446	5738		

Note. CE = compulsive exercise; PE = problematic exercise

**Table 5:** Comparison of Current EDs between current Non-CE group and CE group

		*N* (column %)		Total	χ2	*p*
		Non-CE	CE			
Current EDs	No	1894 (42.09)	100 (8.07)	1994 (34.74)	610.40	< 0.001
	Yes	1918 (42.62)	999 (80.63)	2917 (50.83)		
	Not sure	688 (15.29)	140 (11.30)	828 (14.43)		
EDE-Q cutoff	No	3963 (70.20)	389 (24.09)	4352 (59.94)	1112.28	< 0.001
	Yes	1682 (29.80)	1226 (75.91)	2908 (40.06)		

Note. CE = compulsive exercise; EDs = eating disorders; EDE-Q = Eating Disorders Examination Questionnaire

### Comparison of psychological factors between current non-CE and CE groups

Table 6 presents comparisons of psychological variables, where the CE group scored higher on PHQ-9 depression (d = 0.72), GAD-7 anxiety (d = 0.68), OCI-R obsessive-compulsive traits (d = 0.68), and PHQ-9 item 9 suicidal ideation (d = 0.44). Sensitivity analyses (Table S10) excluding AN yielded similar results (PHQ-9 d = 0.63; GAD-7 d = 0.62; OCI-R d = 0.71), except that suicidal ideation was no longer significantly different (d = 0.28), suggesting that AN participants carried that effect.

**Table 6:** Comparison of psychological factors between the Non-CE and CE groups (Multiple regression, with coviarates age, gender and current BMI)

	CE (M ± SD)		OR (95% CI)	*p*	Phi
	Non-CE (*n* = 5645)	CE (*n* = 1615)			
Depression (PHQ-9)	8.57 ± 6.44	13.27 ± 6.94	1.11 (1.10–1.12)	< 0.001	0.716
Anxiety(GAD-7)	6.58 ± 5.24	10.27 ± 5.90	1.13 (1.11–1.14)	< 0.001	0.684
OCI-R	14.30 ± 11.71	22.67 ± 14.28	1.05 (1.05–1.06)	< 0.001	0.679
Suicidal ideation	0.32 ± 0.71	0.67 ± 1.01	1.62 (1.52–1.72)	< 0.001	0.440

Note. CE = Compulsive exercise; PHQ-9 = Patient Health Questionnaire-9; GAD = Generalized Anxiety Disorder; OCI-R = Obsessive-Compulsive Inventory-Revised

## Discussion

The study included a large clinical sample in which ME was categorized in two ways: presence/absence of lifetime PE and current CE, respectively. The PE group, predominantly female, were younger and had lower BMI compared to the Non-PE group. The PE group were more likely to have AN and less likely to have other EDs, higher lifetime depression and anxiety, and contrary to our expectation, had more often received ED treatment. Current CE was associated with more self-reported ED symptoms and higher levels of depression, anxiety, obsessive-compulsive traits, and suicidal ideation.

### The association between PE and more severe EDs: a complex relationship

Taken together, this suggests that ME signals higher EDs severity and a more complex presentation with other serious psychiatric symptoms being common, much in line with previous findings [48]. As suggested in the sensitivity analyses, this seems particularly salient in the AN group, though many difficulties related to PE were found regardless of ED diagnosis. As to why this may be, several explanations are possible. One line of reasoning suggests individuals who exercise compulsively do so for intrinsic rewards (i.e. exercise is central to their lives and they fear aversive emotions and consequences unless exercising) at the cost of other things in life (relationships, sleep, social activities, occupational functioning), with psychological consequences such as depression, stress, and higher obsessiveness as a result [11, 48, 49]. The higher psychological burden observed in the exercise groups may be an actual consequence of the problematic exercise and what that entails. Another line of reasoning concerns potential deficiencies in treatment; if a core symptom such as exercise persists despite treatment attempts, this may act to maintain EDs and/or increase relapse risk [18]. Longer duration of illness may be associated with greater severity and increased comobidity [50–52]. In addition, repeated experience of treatment non-response may result in hopelessness and demoralization, further curtailing chances of improvement. However plausible these hypotheses may be, our current cross-sectional data do not allow us to test them. A final possible explanation is that problematic exercise and symptoms such as anxiety, depression, obsessions and compulsions are expressions of a shared underlying psychological trait or difficulty, such as perfectionism [53, 54] or emotion dysregulation [55, 56], making them more prone to co-occur. Further research could longitudinally track the trajectory of ED severity and psychiatric comorbidities over time, employing advanced methodologies such as neuroimaging or genetic analyses to elucidate underlying biological mechanisms. Our findings nevertheless underscore the need for comprehensive assessment and tailored treatment interventions informing clinical practice. Thus, exploring the effectiveness of intervention strategies, such as cognitive-behavioral therapy augmented with mindfulness techniques or self-compassion strategies, or further in the future, pharmacological treatments targeting specific neurobiological pathways, could provide valuable insights into how these difficulties are best addressed.

### Diverse treatment preferences for EDs and the need for tailored interventions

Our findings indicate a disparity in treatment-seeking behavior between ED patients with and without PE, with a significantly higher proportion of the PE group having received treatment, specifically medical, psychiatric, and ED inpatient care, as well as care in treatment homes, partial hospitalization, and intensive outpatient treatment. These effects were very small and future research should investigate this with a more tailored design, such as studying how clinicians approach maladaptive exercise and how patients experience treatment in relation to their exercise difficulties. Speculatively however, such treatment-seeking behavior might reflect complex psychological symptoms discussed above: more difficulties may be the reason for needing more care. It also seems related to the fact that AN is more common in this group, although intensive outpatient treatment was more common in the PE group also when removing AN cases. PE when in negative energy balance or underweight might be especially likely to lead to problems that are detected in health care and lead to repeated treatment attempts.

ED severity and psychiatric comorbidity have been highlighted as positively predicting treatment seeking in some studies [57]. However, higher levels of ED cognitions have also been associated with less favorable attitudes toward treatment-seeking [58], and comorbidity has failed to predict treatment-seeking in two large-scale studies [59, 60]. Hence, the severe and complex symptom pattern may contribute to the treatment seeking in the exercise group, but a question raised in addition to this is whether the treatments received are sufficiently effective. If a central symptom (problematic exercise) is not being fully treated it may maintain other difficulties too, and require the individual to keep trying to get adequate treatment.

Studies showing that untreated problematic exercise is related to poorer prognosis and higher risk of relapse in EDs point in a similar direction [12, 13, 18]: something is missing to enable full recovery. On the other hand, individuals with PE were not more likely to report difficulties in finding adequate treatment, so even though they received more treatment, they subjectively rated treatment overall as adequate. Research is needed to explore what these individuals need to achieve sustainable recovery, in order to avoid repeated treatment attempts without substantial improvement and alleviate societal costs. Currently, evidence-based ED treatment manuals offer limited or no specific attention to physical activity. Aditionally, the shortage of physiotherapists and psychologists who are well-versed in the complexities of CE is a pressing concern. To bridge this gap, training for health care providers is needed to better recognize and address this issue, as are evidence-based interventions that cater to the distinct needs of EDs patients with CE. Future research should prioritize the development of tailored interventions, such as “the compulsive exercise activity therapy (LEAP)” [28, 61] designed for patients for whom exercise is part of their EDs symptomatology. LEAP is a cognitive behavioral group therapy offered as a complement to standardized ED treatment. It is delivered conjointly by a psychologist and physiotherapist and focuses on educating patients about CE and how it is maintained, challenging maladaptive beliefs and behaviors, and equipping patients with strategies to promote healthy exercise [28]. If effective, this type of intervention may offer a cost-efficient way to meet an evident treatment need in this population.

### Conceptualizing exercise attitudes and behaviors

Finally, we found an apparent discrepancy between measures; almost a third of the CE group denied having exercised to affect body weight or shape in the past 28 days on the EDE-Q (Table S3). However, the EDE-Q item asks about recent behaviors whereas the CET queries attitudes, which may be present even though the behavior that they concern is currently absent. Nonetheless, future research could investigate whether the attitudinal aspects are predictive of future exercise behavior; a large majority of our sample had exercised in a problematic manner and were therefore presumably at risk of relapsing into such habits. Further, the CE group more often rated themselves as currently having a current ED, whereas degree of match between EDE-Q cutoff and the CET was similar in the CE and Non-CE groups. That is, the CET cutoff carried similar information value relative to the EDE-Q cutoff in both groups, but the subjective rating of current illness differed widely. The CET and the EDE-Q are both based on the cognitive behavioral model of ED [30, 33], and conceptual overlap may account for the better match relative to the EDE-Q. The subjective rating difference may suggest that for individuals scoring above CET cutoff, CE may be assumed to be a central part of their EDs symptoms whereas Non-CE individuals were better characterized by other symptoms domains. This may explain why Non-CE membership was less informative regarding subjective illness experience. The observed discrepancy, if replicated, may also be of value for informing diagnostic criteria involving exercise as different groups may be captured depending on whether behaviour or attitude is primarily assessed.

### Limitations

First, due to the cross-sectional nature of our study, we cannot establish the precise timing of treatment initiation or the onset of PE among participants, limiting our ability to discern causality or temporal relationships. We were also unable to take into account the timing and nature of PE (e.g., information regarding type, detailed frequency, and duration), factors that may be important and would be interesting to study in future research. Second, the dataset, sourced from the EDGI study, has a notable preponderance of AN cases, potentially introducing bias. Sensitivity analyses (i.e., removing AN cases) were conducted however, showing no substantive differences in results compared to full sample analyses except for types of treatment received and suicidal ideation. Again also, effect sizes were limited for lifetime findings and these results need to be replicated and explored further. Although power was limited for treatment types that had been accessed by relatively few participants, it is possible that individuals with a history of AN, normally requiring more treatment than other ED subtypes, carried these effects, suggesting that individuals with AN and PE specifically may access a wider range and frequency of interventions, thereby also driving costs. Suicidal ideation may similarly be associated with CE in individuals with AN history particularly, since this effect became nonsignificant when AN participants were excluded. Conversely, not engaging in CE appears protective against suicidality in AN, as the mean for the Non-CE group increased the most when AN participants were excluded. Additional questions raised by our findings include trajectories of CE symptoms over time and their relationship to other ED symptoms, which could be important to gain actionable clinical insight into how CE affects illness course. A factor that could have affected our results is if CE symptoms were addressed clinically among participants who had undergone ED treatment, but we lacked such data. Again however, CE is not systematically targeted in standardized ED treatment, thus we expect that this had minor influence on findings. Finally, our study relies on self-reported treatment consumption data. We do not have access to objective measures of the specific treatments received by participants.

## Conclusion

The current study found that exercise as part of ED symptomatology was common, with current clinical levels of CE being present in over a fifth of participants, and PE/CE being associated with symptom severity, comorbidity, and poorer outcome over time. This relatively neglected symptom domain is in urgent need of further research and clinical development, and should be assessed thouroughly in terms of both attitudes and behaviors, when working with EDs patients. Further, future versions of diagnostic schemes could consider giving ME a more pronounced description and a more central place, to direct attention and resources toward better management of this impactful symptom domain.

### Electronic supplementary material

Below is the link to the electronic supplementary material.

Supplementary Material 1

## Data Availability

Data is avalaible upon request.

## Abbreviations

ED: Eating Disorder
BED: Binge Eating Disorder
AN: Anorexia Nervosa
CE: Compulsive Exercise
ME: Maladaptive Exercise
EDGI: Eating Disorders Genetics Initiative
EDGI-SE: Eating Disorders Genetics Initiative- Sweden
PE: Problematic Exercise
ED100K: The Eating Disorders 100 000 Questionnaire
EDE-Q: The Eating Disorder Examination Questionnaire
CET: The Compulsive Exercise Test
PHQ-9: The Patient Health Questionnaire-9
GAD-7: The Generalized Anxiety Disorder 7-item scale
OCI-R: The Obsessive-Compulsive Inventory-revised
DSM-5: The Diagnostic and Statistical Manual of mental dDsorders- version 5
OSFED: Other Specified Feeding and Eating Disorders
SCID: The Structured Clinical Interview for DSM-5
LEAP: The compuLsive Exercise Activity theraPy

## References

[1] van Hoeken D, Hoek HW (2020). Review of the burden of eating disorders: mortality, disability, costs, quality of life, and family burden. Curr Opin Psychiatry.

[2] Silén Y, Keski-Rahkonen A (2022). Worldwide prevalence of DSM-5 eating disorders among young people. Curr Opin Psychiatry.

[3] Keski-Rahkonen A, Mustelin L. Epidemiology of eating disorders in Europe: prevalence, incidence, comorbidity, course, consequences, and risk factors. 29, Curr Opin Psychiatry. 2016. 10.1097/YCO.0000000000000278 27662598

[4] Zipfel S, Schmidt U, Giel KE. The hidden burden of eating disorders during the COVID-19 pandemic. 9, Lancet Psychiatry. 2022. 10.1016/S2215-0366(21)00435-1 PMC8673860 34921799

[5] Taquet M, Geddes JR, Luciano S, Harrison PJ (2022). Incidence and outcomes of eating disorders during the COVID-19 pandemic. Br J Psychiatry.

[6] Devoe J, Han D, Anderson A, Katzman A, Patten DK, Soumbasis SB (2023). The impact of the COVID-19 pandemic on eating disorders: a systematic review. Int J Eat Disord.

[7] de Oliveira C, Tanner B, Colton P, Kurdyak P. Understanding the scope of preventable acute care spending among patients with eating disorders. Int J Eat Disord. 2023;56(6). 10.1002/eat.23910 36757092

[8] Bryant E, Spielman K, Le A, Marks P, Aouad P, Barakat S (2022). Screening, assessment and diagnosis in the eating disorders: findings from a rapid review. J Eat Disord.

[9] Kalindjian N, Hirot F, Stona AC, Huas C, Godart N. Early detection of eating disorders: a scoping review [Internet]. Vol. 27, Eating and Weight Disorders. Springer International Publishing; 2022. 21–68 p. 10.1007/s40519-021-01164-x 10.1007/s40519-021-01164-x 33755937

[10] Allen KL, Mountford VA, Elwyn R, Flynn M, Fursland A, Obeid N et al. A framework for conceptualising early intervention for eating disorders. Eur Eat Disord Rev. 2023;31(2). 10.1002/erv.2959 PMC10100476 36426567

[11] Schlegl S, Dittmer N, Hoffmann S, Voderholzer U. Self-reported quantity, compulsiveness and motives of exercise in patients with eating disorders and healthy controls: differences and similarities. J Eat Disord. 2018;6(1). 10.1186/s40337-018-0202-6 PMC6038234 30002829

[12] Levallius J, Collin C, Birgegård A. Now you see it, now you don’t: compulsive exercise in adolescents with an eating disorder. J Eat Disord. 2017. 10.1186/s40337-016-0129-8 PMC5376699 28392917

[13] Monell E, Levallius J, Forsén Mantilla E, Birgegård A (2018). Running on empty - a nationwide large-scale examination of compulsive exercise in eating disorders. J Eat Disord.

[14] Rizk M, Mattar L, Kern L, Berthoz S, Duclos J, Viltart O et al. Physical activity in eating disorders: a systematic review. Nutrients. 2020;12(1). 10.3390/nu12010183 PMC7019575 31936525

[15] Dalle Grave R, Calugi S, Marchesini G (2008). Compulsive exercise to control shape or weight in eating disorders: prevalence, associated features, and treatment outcome. Compr Psychiatry.

[16] Meyer C, Plateau CR, Taranis L, Brewin N, Wales J, Arcelus J. The compulsive Exercise Test: confirmatory factor analysis and links with eating psychopathology among women with clinical eating disorders. J Eat Disord. 2016;4(1). 10.1186/s40337-016-0113-3 PMC4992271 27547403

[17] Solenberger SE. Exercise and eating disorders: a 3-year inpatient hospital record analysis. Eat Behav. 2001;2(2). 10.1016/s1471-0153(01)00026-5 15001043

[18] Carter JC, Blackmore E, Sutandar-Pinnock K, Woodside DB (2004). Relapse in anorexia nervosa: a survival analysis. Psychol Med.

[19] Noetel M, Miskovic-Wheatley J, Crosby RD, Hay P, Madden S, Touyz S. A clinical profile of compulsive exercise in adolescent inpatients with anorexia nervosa. J Eat Disord [Internet]. 2016;4(1):1–10. 10.1186/s40337-016-0090-6 10.1186/s40337-016-0090-6 PMC4744387 26855778

[20] Meyer C, Taranis L (2011). Exercise in the eating disorders: terms and definitions. Eur Eat Disord Rev.

[21] Dittmer N, Jacobi C, Voderholzer U. Compulsive exercise in eating disorders: proposal for a definition and a clinical assessment. J Eat Disord. 2018;6(1). 10.1186/s40337-018-0219-x PMC6260729 30505444

[22] Scharmer C, Martinez K, Gorrell S, Reilly EE, Donahue JM, Anderson DA (2020). Eating disorder pathology and compulsive exercise during the COVID-19 public health emergency: examining risk associated with COVID-19 anxiety and intolerance of uncertainty. Int J Eat Disord.

[23] Fietz M, Touyz S, Hay P. A risk profile of compulsive exercise in adolescents with an eating disorder: a systematic review. Adv Eat Disord. 2014;2(3).

[24] Cosh SM, McNeil DG, Tully PJ. Compulsive exercise and its relationship with mental health and psychosocial wellbeing in recreational exercisers and athletes. J Sci Med Sport. 2023;26(7). 10.1016/j.jsams.2023.05.006 37296060

[25] Edlund K, Johansson F, Lindroth R, Bergman L, Sundberg T, Skillgate E. Body image and compulsive exercise: are there associations with depression among university students? Eat Weight Disord. 2022;27(7). 10.1007/s40519-022-01374-x PMC9556381 35179726

[26] Szabo A, Demetrovics Z, Griffiths MD. Morbid exercise behavior: Addiction or psychological escape? In: The Exercise Effect on Mental Health: Neurobiological Mechanisms. 2018.

[27] Shroff H, Reba L, Thornton LM, Tozzi F, Klump KL, Berrettini WH et al. Features associated with excessive exercise in women with eating disorders. Int J Eat Disord. 2006;39(6). 10.1002/eat.20247 16637047

[28] Monell E, Meyer C, Szwajda A, Forsén Mantilla E. Taking the LEAP: study protocol for a randomized, multicentre, naturalistic, efficacy trial of the compuLsive Exercise Activity theraPy (LEAP) - a cognitive behavioral program specifically targeting compulsive exercise in patients with eating disorders. BMC Psychiatry. 2021;21(1). 10.1186/s12888-021-03356-2 PMC8299452 34301226

[29] American Psychiatric Association. DSM-5. DIAGNOSTIC STATISTICAL MANUAL OF MENTAL DISORDER. Volume 5. American Psychiatric Association; 2013.

[30] Taranis L, Touyz S, Meyer C. Disordered eating and exercise: development and preliminary validation of the compulsive exercise test (CET). Eur Eat Disord Rev. 2011;19(3). 10.1002/erv.1108 21584918

[31] Bulik CM, Thornton LM, Parker R, Kennedy H, Baker JH, MacDermod C et al. The Eating disorders Genetics Initiative (EDGI): study protocol. BMC Psychiatry. 2021;21(1). 10.1186/s12888-021-03212-3 PMC8097919 33947359

[32] https://www.ipsos.com/sv-se [Internet]. 2024. IPSOS.

[33] Fairburn CG, Beglin SJ, Fairburn CG (2008). Eating disorders Examination Questionnaire (EDE-Q 6.0). Cognitive behavior therapy and eating disorders.

[34] Kroenke K, Spitzer RLL, Williams JBB, Kroenke K, Spitzer RLL, Williams JBB. The PHQ-9: validity of a brief depression screening measure. J Gen Intern Med. 2001;16. 10.1046/j.1525-1497.2001.016009606.x PMC1495268 11556941

[35] Spitzer RL, Kroenke K, Williams JBW, Löwe B. A brief measure for assessing generalized anxiety disorder: the GAD-7. Arch Intern Med. 2006;166(10). 10.1001/archinte.166.10.1092 16717171

[36] Foa EB, Huppert JD, Leiberg S, Langner R, Kichic R, Hajcak G et al. The obsessive-compulsive inventory: development and validation of a short version. Psychol Assess. 2002;14(4). 12501574

[37] First MB, Williams JBW, Karg RS, Spitzer RL. Structured clinical interview for DSM-5 disorders, Clinician Version (SCID-5-CV). Am Psychiatric Association. 2016.

[38] Thornton LM, Munn-Chernoff MA, Baker JH, Juréus A, Parker R, Henders AK et al. The Anorexia Nervosa Genetics Initiative (ANGI): overview and methods. Contemp Clin Trials. 2018;74. 10.1016/j.cct.2018.09.015 PMC6338222 30287268

[39] Velkoff EA, Brown TA, Kaye WH, Wierenga CE. Using clinical cutoff scores on the eating disorder examination-questionnaire to evaluate eating disorder symptoms during and after naturalistic intensive treatment. Eat Disord. 2023;31(5). 10.1080/10640266.2023.2191488 36935579

[40] Luce KH, Crowther JH (1999). The reliability of the eating disorder examination - self-report Questionnaire Version (EDE-Q). Int J Eat Disord.

[41] Peterson CB, Crosby RD, Wonderlich SA, Joiner T, Crow SJ, Mitchell JE et al. Psychometric properties of the eating disorder examination-questionnaire: factor structure and internal consistency. Int J Eat Disord. 2007;40(4). 10.1002/eat.20373 17304585

[42] Kocalevent RD, Hinz A, Brähler E. Standardization of the depression screener Patient Health Questionnaire (PHQ-9) in the general population. Gen Hosp Psychiatry. 2013;35(5). 10.1016/j.genhosppsych.2013.04.006 23664569

[43] Beard C, Hsu KJ, Rifkin LS, Busch AB, Björgvinsson T. Validation of the PHQ-9 in a psychiatric sample. J Affect Disord. 2016;193. 10.1016/j.jad.2015.12.075 26774513

[44] Hinz A, Klein AM, Brähler E, Glaesmer H, Luck T, Riedel-Heller SG et al. Psychometric evaluation of the generalized anxiety disorder screener GAD-7, based on a large German general population sample. J Affect Disord. 2017;210. 10.1016/j.jad.2016.12.012 28088111

[45] Löwe B, Decker O, Müller S, Brähler E, Schellberg D, Herzog W et al. Validation and standardization of the generalized anxiety disorder screener (GAD-7) in the general population. Med Care. 2008;46(3). 10.1097/MLR.0b013e318160d093 18388841

[46] Wootton BM, Diefenbach GJ, Bragdon LB, Steketee G, Frost RO, Tolin DF. A contemporary psychometric evaluation of the obsessive compulsive inventory-revised (OCI-R). Psychol Assess. 2015;27(3). 10.1037/pas0000075 PMC4530108 25664634

[47] Huppert JD, Walther MR, Hajcak G, Yadin E, Foa EB, Simpson HB et al. The OCI-R: validation of the subscales in a clinical sample. J Anxiety Disord. 2007;21(3). 10.1016/j.janxdis.2006.05.006 16814981

[48] Lichtenstein MB, Hinze CJ, Emborg B, Thomsen F, Hemmingsen SD. Compulsive exercise: links, risks and challenges faced. Psychol Res Behav Manag. 2017;Volume 10. 10.2147/PRBM.S113093 PMC5386595 28435339

[49] Weinstein A, Weinstein Y (2014). Exercise Addiction- Diagnosis, Bio-psychological mechanisms and Treatment issues. Curr Pharm Des.

[50] Lin JA, Jhe G, Vitagliano JA, Milliren CE, Spigel R, Woods ER et al. The Association of Malnutrition, illness duration, and pre-morbid weight status with anxiety and depression symptoms in adolescents and young adults with restrictive eating disorders: a cross-sectional study. J Eat Disord. 2021;9(1). 10.1186/s40337-021-00415-7 PMC8127488 34001260

[51] Takakura S, Aso CS, Toda K, Hata T, Yamashita M, Sudo N. Physical and psychological aspects of anorexia nervosa based on duration of illness: a cross-sectional study. Biopsychosoc Med. 2019;13(1). 10.1186/s13030-019-0173-0 PMC6929428 31889996

[52] Andrés-Pepiñá S, Plana MT, Flamarique I, Romero S, Borràs R, Julià L et al. Long-term outcome and psychiatric comorbidity of adolescent-onset anorexia nervosa. Clin Child Psychol Psychiatry. 2020;25(1). 10.1177/1359104519827629 30764636

[53] Cresswell C, Watson HJ, Jones E, Howell JA, Egan SJ. The role of compulsive exercise in the relationship between perfectionism and eating disorder pathology in underweight adolescents with eating disorders. Eat Behav. 2022;47. 10.1016/j.eatbeh.2022.101683 36410135

[54] Egan SJ, Bodill K, Watson HJ, Valentine E, Shu C, Hagger MS. Compulsive exercise as a mediator between clinical perfectionism and eating pathology. Eat Behav. 2017;24. 10.1016/j.eatbeh.2016.11.001 27865202

[55] Cuesta-Zamora C, González-Martí I, García-López LM, Ros L, Plateau CR, Ricarte JJ. Emotion dysregulation as a mediator of the relationship between anxiety, compulsive exercise and eating disorder symptoms in adolescents. Children. 2021;8(12). 10.3390/children8121088 PMC8700564 34943286

[56] Forsén Mantilla E, Clinton D, Monell E, Levallius J, Birgegård A (2022). Impulsivity and compulsivity as parallel mediators of emotion dysregulation in eating-related addictive-like behaviors, alcohol use, and compulsive exercise. Brain Behav.

[57] Ali K, Farrer L, Fassnacht DB, Gulliver A, Bauer S, Griffiths KM. Perceived barriers and facilitators towards help-seeking for eating disorders: a systematic review. Int J Eat Disord. 2017. 10.1002/eat.22598 27526643

[58] Dotson KB, Masuda A, Cohen LL. Disordered eating cognitions as predictors of attitudes toward seeking Professional Psychological services. Int J Adv Couns. 2011;33(4).

[59] Forrest LN, Smith AR, Swanson SA (2017). Characteristics of seeking treatment among U.S. adolescents with eating disorders. Int J Eat Disord.

[60] Bohrer BK, Carroll IA, Forbush KT, Chen PY. Treatment seeking for eating disorders: results from a nationally representative study. Int J Eat Disord. 2017;50(12). 10.1002/eat.22785 28963793

[61] Taranis L, Touyz S, La Puma M, Meyer C. Loughborough eating-disorders activity Programme LEAP group cognitive-behavioural therapy for compulsive exercise in the eating disorders: therapist manual. Loughborough University; 2011.

