# Supplementary material for: Maladaptive exercise in eating disorders: lifetime and current impact on mental health and treatment seeking
Source: J Eat Disord. 2024 Jun 24;12:86. doi: 10.1186/s40337-024-01048-2 (PMC11194861; doi:10.1186/s40337-024-01048-2)
Supplement: Supplementary file 1 — Supplementary Material 1 [file 40337_2024_1048_MOESM1_ESM.docx]

Supplementary tables

Sensitivity Table S1. *Demographic and clinical characteristics for the Non-PE and PE groups without AN cases*

| Category | | *N* (column %) or *M*±*SD* | | Total | χ^2^ or *t* | *p* |
| --- | --- | --- | --- | --- | --- | --- |
|  |  | Non-PE | PE |  |  |  |
| Gender | Male | 34 (7.34) | 53 (3.63) | 87 (4.52) | 12.027 | 0.007 |
|  | Female | 420 (90.71) | 1388 (94.94) | 1808 (93.92) |  |  |
|  | Non-binary | 8 (1.73) | 19 (1.30) | 27 (1.40) |  |  |
|  | Others | 1 (0.22) | 2 (0.14) | 3 (0.16) |  |  |
| Diagnosis | AN | 0 (0.00) | 0 (0.00) | 0 (0.00) |  |  |
|  | BN | 90 (23.44) | 648 (50.12) | 738 (44.01) | 146.841 | <0.001 |
|  | BED | 81 (21.09) | 62 (4.80) | 143 (8.53) |  |  |
|  | OSFED | 213 (55.47) | 583 (45.09) | 796 (47.47) |  |  |
| Compensation |  | 367 (79.27) | 1415 (96.79) | 1782 (90.69) | 154.41 | <0.001 |
| Current ED |  | 157 (69.16) | 560 (53.85) | 717 (56.59) | 17.799 | <0.001 |
| ED treatment |  | 235 (50.76) | 1064 (72.78) | 1299 (67.48) | 77.706 | <0.001 |
| Age | | 41.39±13.07 | 32.60±9.87 | 34.72±11.36 | 13.311 | <0.001 |
| Age at first symptom | | 18.59±7.20 | 15.48±4.40 | 16.23±5.38 | 8.765 | <0.001 |
| Current BMI | | 30.73±8.30 | 26.50±5.74 | 27.52±6.70 | 10.223 | <0.001 |
| EDEQ score | | 2.47±1.46 | 2.73±1.59 | 2.67±1.56 | -3.305 | 0.001 |

Note. PE=problematic exercise; AN=anorexia nervosa; BN=bulimia nervosa; BED=binge eating disorder; OSFED=other specified feeding or eating disorder; EDs=eating disorders; BMI=body mass index; EDEQ=eating disorders examination questionnaire.

Sensitivity Table S2. *Demographic and clinical characteristics of Non-CE group and CE groups without AN cases*

| Category | | *N* (column %) or *M*±*SD* | | Total | χ^2^ or *t* | *p* |
| --- | --- | --- | --- | --- | --- | --- |
|  |  | Non-CE | CE |  |  |  |
| Gender | Male | 73(3.89) | 30(6.20) | 103(4.37) | 5.966 | 0.113 |
|  | Female | 1781(94.99) | 446(92.15) | 2227(94.40) |  |  |
|  | Non-binary | 19(1.01) | 7(1.45) | 26(1.10) |  |  |
|  | Others | 2(0.11) | 1(0.21) | 3(0.13) |  |  |
| Diagnosis | AN | 0(0.00) | 0(0.00) | 0(0.00) | 20.909 | <0.001 |
|  | BN | 681(42.01) | 213(48.41) | 894(43.38) |  |  |
|  | BED | 174(10.73) | 17(3.86) | 191(9.27) |  |  |
|  | OSFED | 766(47.25) | 210(47.73) | 976(47.36) |  |  |
| Compensation |  | 570(30.40) | 244(50.41) | 814(34.51) | 71.41 | <0.001 |
| Current ED |  | 631(51.85) | 224(78.87) | 855(56.96) | 75.021 | <0.001 |
| ED treatment |  | 1253(66.83) | 291(60.12) | 1544(65.45) | 7.643 | 0.006 |
| Age | | 35.30±11.25 | 33.45±10.85 | 34.92±11.19 | 3.237 | 0.001 |
| Age at first symptom | | 16.16±5.28 | 15.85±5.24 | 16.10±5.28 | 1.166 | 0.244 |
| Current BMI | | 27.61±6.60 | 26.66±6.38 | 27.41±6.57 | 2.895 | 0.004 |
| EDE-Q Total | | 2.37±1.47 | 3.89±1.17 | 2.68±1.54 | -24.061 | <0.001 |

Note. CE=compulsive exercise; AN=anorexia nervosa; BN=bulimia nervosa; BED=binge eating disorder; OSFED=other specified feeding or eating disorder; EDs=eating disorders; BMI=body mass index; EDE-Q=eating disorders examination questionnaire.

Sensitivity Table S3. *Comparison of treatment types for EDs between the Non-PE group and PE group without AN cases*

|  | *N* (column %) | | Total | χ^2^ | *p* |
| --- | --- | --- | --- | --- | --- |
| Type of treatment | Non-PE | PE |  |  |  |
| Medical ward inpatient care | 6(2.69) | 15(1.46) | 21(1.68) | 1.678 | 0.195 |
| Psychiatric inpatient care | 11(4.93) | 39(3.80) | 50(4.00) | 0.615 | 0.433 |
| Inpatient care at an eating disorder unit | 9(4.04) | 53(5.16) | 62(4.96) | 0.492 | 0.483 |
| Care in treatment homes | 3(1.35) | 24(2.34) | 27(2.16) | 0.852 | 0.356 |
| Day treatment/partial hospitalization | 31(13.90) | 159(15.48) | 190(15.20) | 0.355 | 0.551 |
| Intensive outpatient treatment | 20(8.97) | 164(15.97) | 184(14.72) | 7.152 | 0.007 |
| Outpatient treatment | 160(71.75) | 705(68.65) | 865(69.20) | 0.827 | 0.363 |
| Emergency room visit | 58(26.01) | 264(25.71) | 322(25.76) | 0.009 | 0.925 |
| Others | 9(4.04) | 42(4.09) | 51(4.08) | 0.001 | 0.971 |

Note. PE=problematic exercise.

Supplementary Table S4. *Comparison of therapy received for EDs between the Non-PE group and PE groups*.

|  | Group (column %) | | Total | χ2 | p |
| --- | --- | --- | --- | --- | --- |
|  | Non-PE | PE |  |  |  |
| Family counseling | 35(8.95) | 1058(22.73) | 1093(21.67) | 40.37 | <0.001 |
| Cognitive behavior therapy | 175(44.76) | 2639(56.70) | 2814(55.78) | 20.87 | <0.001 |
| Dialectical behavior therapy | 11(2.81) | 246(5.29) | 257(5.09) | 4.56 | 0.033 |
| Psychodynamic therapy | 23(5.88) | 439(9.43) | 462(9.16) | 5.47 | 0.019 |
| Interpersonal therapy | 5(1.28) | 86(1.85) | 91(1.80) | 0.66 | 0.417 |
| Psychotherapy | 57(14.58) | 989(21.25) | 1046(20.73) | 9.77 | 0.002 |
| Group therapy | 1(0.26) | 17(0.37) | 18(0.36) | 0.12 | 0.727 |
| Physiotherapy (Physical therapy/ body awareness) | 69(17.65) | 1416(30.43) | 1485(29.44) | 28.37 | <0.001 |

Note. PE=problematic exercise.

Sensitivity Table S5. *Comparison of therapy received for EDs between the Non-PE group and PE groups* without AN cases

|  | Group (column %) | | Total | χ2 | p |
| --- | --- | --- | --- | --- | --- |
|  | Non-PE | PE |  |  |  |
| Family counseling | 8(3.59) | 75(7.30) | 83(6.64) | 4.08 | 0.043 |
| Cognitive behavior therapy | 95(42.60) | 557(54.24) | 652(52.16) | 9.939 | 0.002 |
| Dialectical behavior therapy | 7(3.14) | 28(2.73) | 35(2.80) | 0.115 | 0.735 |
| Psychodynamic therapy | 10(4.48) | 77(7.50) | 87(6.96) | 2.569 | 0.109 |
| Interpersonal therapy | 4(1.79) | 23(2.24) | 27(2.16) | 0.172 | 0.678 |
| Psychotherapy | 27(12.11) | 164(15.97) | 191(15.28) | 2.11 | 0.146 |
| Group therapy | 52(23.32) | 269(26.19) | 321(25.68) | 0.793 | 0.373 |
| Physiotherapy (Physical therapy/ body awareness) | 35(15.70) | 185(18.01) | 220(17.60) | 0.679 | 0.41 |

Note. PE=problematic exercise.

Supplementary Table S6. *Comparison of how difficult Non-PE and PE groups found it to access treatment for their ED.*

|  |  | Group (column %) | | Total | χ2 | *p* |
| --- | --- | --- | --- | --- | --- | --- |
|  |  | Non-PE | PE |  |  |  |
| How difficult do you feel it was to get access to adequate treatment? | Not difficult at all | 46 (20.4) | 755(19.2) | 801 | 5.65 | 0.342 |
|  | Easier than for other ailments | 18(8.0) | 443(11.3) | 461 |  |  |
|  | As difficult as for other ailments | 58(25.8) | 925(23.5) | 983 |  |  |
|  | Hard | 50(22.2) | 1010(25.7) | 1060 |  |  |
|  | Very hard | 53(23.56) | 805(20.44) | 858 |  |  |

Note. PE=problematic exercise.

Supplementary Table S7. *Crosstabulation and chi-square of CE grouping and endorsement of exercise on the EDE-Q*

|  |  | Group (column %) | | Total | χ2 | *p* |
| --- | --- | --- | --- | --- | --- | --- |
|  |  | Non-CE | CE |  |  |  |
| In the past 28 days, how many times have you exercised excessively or compulsively to control your weight, figure, amount of fat, or to burn calories? | No | 4400 (77.9) | 496 (30.7) | 4896 | 1274.77 | <0.001 |
|  | Yes | 1247 (22.1) | 1121 (69.3) | 2368 |  |  |
| Total | | 5647 | 1617 | 7264 |  |  |
|  | |  |  |  |  |  |

Note. CE=Compulsive exercise.

Sensitivity Table S8. *Crosstabulation and chi-square of current CE and lifetime PE without AN cases*

|  |  | CE (column %) | | Total | χ^2^ | *p* |
| --- | --- | --- | --- | --- | --- | --- |
|  |  | Non-CE | CE |  |  |  |
| PE | Non-PE | 308(24.25) | 20(4.87) | 328(19.51) | 74.30 | <0.001 |
|  | PE | 962(75.75) | 391(95.13) | 1353(80.49) |  |  |
| Total | | 1270 | 411 | 1681 |  |  |

Note. CE=compulsive exercise; PE=problematic exercise.

Sensitivity Table S9. *Comparison of Current EDs between current Non-CE group and CE group without AN cases*

|  |  | *N* (column %) | | Total | χ^2^ | *p* |
| --- | --- | --- | --- | --- | --- | --- |
|  |  | Non-CE | CE |  |  |  |
| Current ED | No | 410(33.69) | 29(10.21) | 439(29.25) | 75.02 | <0.001 |
|  | Yes | 631(51.85) | 224(78.87) | 855(56.96) |  |  |
|  | Not sure | 176(14.46) | 31(10.92) | 207(13.79) |  |  |
|  |  |  |  |  |  |  |
| EDE-Q cutoff | No | 1113(59.36) | 91(18.80) | 1204(51.04) | 253.24 | <0.001 |
|  | Yes | 762(40.64) | 393(81.20) | 1155(48.96) |  |  |

Note. CE=compulsive exercise; ED=eating disorders; EDE-Q=Eating Disorders Examination Questionnaire.

Sensitivity Table S10. *Comparison of psychological factors between the Non-CE and CE groups (Multiple regression, with coviarates age, gender and current BMI, removing AN cases).*

|  | CE (*M*±*SD*) | | *OR(95%CI)* | *p* |
| --- | --- | --- | --- | --- |
|  | Non-CE (*n*=1875) | CE (*n*=484) |  |  |
| Depression (PHQ-9) | 8.86±6.43 | 12.92±6.50 | 1.10(1.09-1.12) | <0.001 |
| Anxiety(GAD-7) | 6.21±5.13 | 9.50±5.74 | 1.12(1.09-1.14) | <0.001 |
| OCI_R | 13.53±11.03 | 21.70±13.41 | 1.06(1.05-1.06) | <0.001 |
| Suicidal ideation | 0.57±0.50 | 0.60±0.49 | 1.14(0.92-1.43) | 0.237 |

Note. CE=Compulsive exercise; PHQ-9=Patient Health Questionnaire-9; GAD=Generalized Anxiety Disorder; OCI-R=Obsessive-Compulsive Inventory-Revised.
